# The Role of Water Volume Fraction on Water Adsorption in Anion Exchange Membranes

**DOI:** 10.1021/acs.macromol.5c01256

**Published:** 2025-09-11

**Authors:** Gervasio Zaldivar, Ruilin Dong, Joan M. Montes de Oca, Ge Sun, Riccardo Alessandri, Christopher G. Arges, Shrayesh N. Patel, Paul F. Nealey, Juan J. de Pablo

**Affiliations:** † Department of Chemical and Biomolecular Engineering, 2462 Tandon School of Engineering, New York University, New York, Brooklyn 11201, United States; ‡ Department of Computer Science, Courant Institute of Mathematical Sciences, New York University, New York, New York 10012, United States; § Department of Physics, New York University, New York, New York 10003, United States; ∥ Pritzker School of Molecular Engineering, University of Chicago, Chicago, Illinois 60637-1476, United States; ⊥ Materials Science Division, 537448 Argonne National Laboratory, Lemont, Illinois 60439-4801, United States; # Department of Chemical Engineering, KU Leuven, Celestijnenlaan 200J, Leuven 3001, Belgium; ∇ Applied Materials Division, 1291 Argonne National Laboratory, Lemont, Illinois 60439-4801 United States

## Abstract

Water absorption plays a key role in the performance of polymeric anion exchange membranes. It influences important properties such as ionic conductivity and mechanical strength and alters their performance as solid electrolytes in hydrogen electrochemical devices for energy conversion. However, computational approaches that address the relationship between the polymer design and the absorption process are scarce. In this work, we introduce a simple thermodynamic model to predict the water absorption isotherms of polyelectrolyte membranes in contact with a water vapor reservoir that incorporates the specific chemical design of the polymers. The model accurately predicts the water content and macrostructural properties of polynorbornene membranes as a function of the water activity and successfully captures the effect of various polymer design parameters. The energy of pairwise attractive interactions predicted by our model provides a means to interpret the absorption process at the molecular level. The model also reveals the most significant favorable and unfavorable contributions to the free energy and indicates that their balance is solely governed by the water volume fraction, regardless of the polymer design. This universal behavior leads to important implications in the search for better ion exchange membranes.

## Graphical abstract

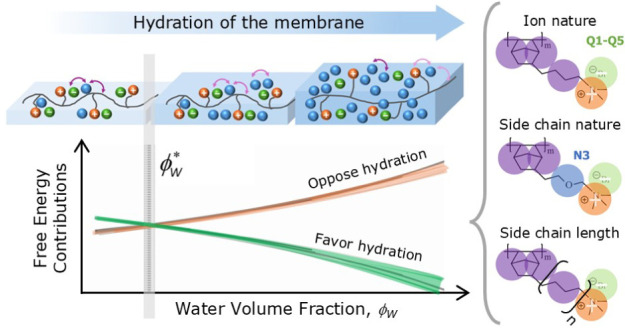

## Introduction

There has been significant growth in anion exchange membrane (AEM) research over the past decade.^1–4^ Because they foster an alkaline environment, they assist in the development of low-cost fuel cells^5,6^ and water electrolyzers.^7–9^ The Faradaic reactions in alkaline environments do not necessitate precious group metals. Furthermore, they prevent metal cation crossover in redox flow batteries,^10^ demonstrating their broad utility to several types of electrochemical energy storage and conversion technologies.

AEMs are made from polymeric materials composed of a nonpolar matrix with bound cationic groups and mobile negative counterions. This structure allows for the transport of mobile anions through a solid membrane while providing electronic insulation for the electrodes. Although the performance of commercial AEMs is rapidly approaching that of PEMs, they continue to face challenges due to their comparatively inferior chemical stability and ion conductivity.^11,12^

Membrane hydration is central to overcoming the said challenges. During operation, the membranes absorb water from air due to the hydrophilic character of the ions, which significantly affects the properties of the material. In particular, water plays a relevant role in the ionic conductivity of the membrane. The ionic conductivity of AEMs and PEMs is low in the dry state and becomes significant only above a certain water content.^7,13–15^ For example, in recent work from our group,^16^ we showed that polynorborene anion exchange membranes become significantly more conductive at water contents above ∼3 water molecules per ion pair. In addition, the presence of water may have a positive impact on the alkaline stability of the material.^17,18^ However, water can also have detrimental effects. An excessive amount of water results in materials with poor mechanical properties. Specifically, the Young’s modulus and breaking stress of the membranes decrease with water content.^14,19,20^ Hence, excess water uptake in a membrane makes it unsuitable as a separator in electrochemical devices involving water and humidity.

Understanding water absorption is necessary for the design of novel membranes. From an experimental perspective, membrane hydration is determined from the sorption isotherms of the materials in equilibrium with a humid environment, that is, plots of the water content as a function of the relative humidity of the reservoir at a fixed temperature.^19,21–23^ From a theoretical perspective, calculating isotherms from particle-based computational simulations presents difficulties. Past work in the literature has been restricted to a narrow polymer design space and often limited to the high-water-activity regime.^24–27^

As an alternative, theoretical approaches capable of directly describing the system’s averaged equilibrium structure through approximations can be useful tools with which to generate structural and thermodynamic information about the membranes, including water sorption isotherms.^28–31^ This can be achieved at much lower computational demands than particle-based simulations, but at the expense of losing a description of the instantaneous states of the system and its fluctuations. Moreover, the incorporation of specific molecular designs in this kind of framework can be challenging. Although several examples of macroscopic models and self-consistent mean-field models are available for the study of water and ion uptake in membranes^31–34^ the development of general approaches for the absorption of water in ion-exchange membranes based on molecular-scale physical phenomena remains an open challenge.

In this work, we present a simple mean-field approach to predict the equilibrium absorption isotherms of ion-exchange membranes. The model is a homogeneous version of the one presented in ref 35 for nonpolar solvent isotherms of polymer-decorated nanoparticle superlattices and is extended here to consider water as a solvent and richer polymer chemistry. Despite the simplicity of the model, its inputs are molecular properties rather than free-fitting parameters or empirical macroscopic properties. Furthermore, the polymer molecular model is based on the Martini 3 coarse-grained force field,^36^ which makes it easily transferable to other chemical designs in addition to the ones considered in this study. Our focus is to develop a model that investigates the absorption phenomenon from a general fundamental perspective that will allow us to explore a wide polymer design space with the aim of finding general trends that can guide the development of new materials.

To validate the model, we compare its structural and thermodynamic predictions with experimental measurements and molecular dynamic simulations for polynorbornene membranes with different degrees of functionalization. Polynorbornenes provide a great platform for exploring the chemical design of the matrix because of their electrochemically stable backbone and easily functionalizable side chains.^16^ We also study the effect of different design parameters that are commonly explored in the literature; namely, the nature of the counterion as well as the side chain hydrophobicity and length. Our predictions for the effect of these parameters on the water absorption process provide further validation by comparison with reported experimental results. Moreover, from the analysis of the thermodynamics of the hydration process for the various polymer designs considered, the model reveals universal behaviors with important implications in the development of novel ion exchange membranes.

## Theoretical Methods

We consider a bulk AEM in contact with a water vapor reservoir at fixed vapor pressure *p*_*vap*_, i.e., fixed water activity $a_{w}=p_{vap}/p_{vap}^{*}$ , where *p*_*vap*_ is the reservoir pressure and *p* ^*^_vap_ is the saturation vapor pressure. A schematic representation of the system is provided in Figure 1. We investigated AEMs made from polynorbornenes, whose chemical structure is shown in the bottom of Figure 1, for different degrees of functionalization, i.e., *DoF* = 100% *n*_*charged*_/(*n*_*charged*_ + *n*_*neutral*_) where *n*_*charged*_ and *n*_*neutral*_ are the number of charged and neutral monomers on each chain, respectively. We also consider a wider design space to address the effects of the counterion nature, side chain length, and hydrophobicity. All species in the system are modeled at a coarse-grained level, where each bead approximately represents 4 atoms; see Figure 1. More details of the molecular model can be found in the Supporting Information.

**Figure 1:**
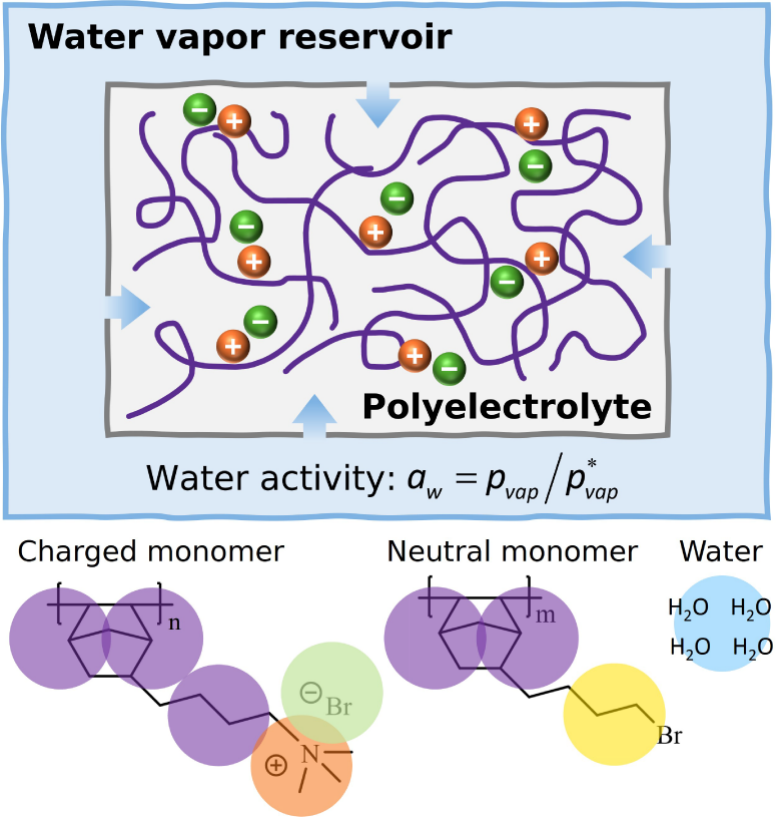
Schematic representation of the system considered in this work (top). Chemical structure of the monomers that form the polynorbornene studied in this work, and their corresponding coarse-grained mapping (bottom).

The goal of the model is to obtain the equilibrium water content of the system for a given water activity. The approach consists of proposing a free-energy density expression for the system and finding its minimum, which ultimately leads to equilibrium thermodynamic and structural information as a function of water activity. The procedure is described in detail in the SI and is only briefly addressed in what follows.

For a system with *N*_*p*_ polymer chains in a volume *V* at temperature *T* = (*k*_B_β)^−1^(where *k*_*B*_ is the Boltzmann constant) in contact with a water vapor reservoir with fixed water activity, i.e., fixed chemical potential μ_*w*_, the free energy density is given by

1 $$\frac{\beta \Omega \left(N_{p},V,T,\mu_{w}\right)}{V}=\beta \omega \left(\rho_{p},T,\mu_{w}\right)=\beta F_{tr}+\beta F_{vdw}+\beta F_{HS}+\beta F_{IP}+\beta F_{born}-\rho_{w}\mu_{w}$$

where ρ_*p*_ and ρ_*w*_ are the number densities of polymer chains and water beads, respectively. Ω is a thermodynamic potential that is canonical for polymer chains and grand canonical for water. Each term in eq 1 contributes to the free energy; namely, β*F*_*tr*_ is the free energy related to the translational entropy of the species in the system, β*F*_*vdw*_ is the energy due to short-range attractions between beads, modeled through an integrated Lennard-Jones attractive potential with parameters taken from the nonbonded interactions of the Martini 3 force field,^36^ β*F*_*HS*_ is the energy due to the hard-sphere interbead repulsions, given by the Carnhan-Starling equation of state,^35,37^ β*F*_*IP*_ is the free energy associated with the possibility that charged beads in the polymer chain and counterions form ion pairs, and β*F*_*born*_ is the self-energy of ions^38^ that controls the intrinsic strength of the ion pair formation. Detailed expressions for each term are presented in the SI.

Note that the system is considered to be homogeneous and neutral; hence, we do not explicitly consider electrostatic interactions. However, since it has been shown that the ion–ion short-range correlations can be important in polyelectrolyte systems, we also include the ion-pairing energy as a way to recover such correlations at a mean-field level. This approach has been widely applied to the study of polyelectrolytes in solution.^39–42^ We will show later that this term ultimately represents a minor contribution to the water absorption process.

Another approximation of the model is that it does not consider chain connectivity, i.e., the free energy density does not include a contribution from the internal degrees of freedom of the polymer chains. Note that chain correlation effects in polyelectrolyte systems have been discussed before by Qin and de Pablo,^43^ Fredrickson^44^ and Olvera de la Cruz^45^ among others. Such effects are not expected to alter the qualitative behavior of our highly coarse-grained model and they are not included in this work for simplicity.

We calculate the water absorption isotherms using the following procedure. We minimize the free energy density *βω* with respect to the number density of water ρ_*w*_ and the fraction of charged monomers that do not form ion pairs *f*. As a result, we obtain expressions for ρ_*w*_ and *f* that together form a set of coupled nonlinear equations that are solved numerically. To find the equilibrium properties, we calculate the excess minimized free energy density per chain relative to the water vapor reservoir, *βω* ^*ex*^, for a given water activity and polymer chain number density, ρ_*p*_ (i.e., a given set of natural system variables: ρ_*p*_, *T*, and μ_*w*_). The equilibrium ρ_*p*_ is the value that minimizes *βω* ^*ex*^. This is equivalent to determining the volume that equalizes the pressure between the system and the reservoir for a fixed number of polymer chains and constant water activity (see SI and ref 35). The water content for each given water activity is then calculated using the equilibrium values of ρ_*p*_ and ρ_*w*_. For example, the hydration number (number of water molecules per ion pair) is given by λ = 4ρ_*w*_/(ρ*_p_n*_*charged*_), where 4 represents the number of water molecules included in a coarse-grained water bead.

## Results

### Water Content, Volume Expansion, and Ion Concentration

The model accurately captures the behavior of absorption isotherms for polymers with varying degrees of functionalization. Figure 2a shows the model’s prediction for the water content (expressed as hydration number, i.e., number of water molecules per ion pair) in comparison to experimental measurements for the same system (sourced from ref 16). The predictions of the model are in agreement with experiments, effectively capturing the effect of the degree of functionalization of the polymer. As expected, the water content decreases as the degree of functionalization decreases.

**Figure 2:**
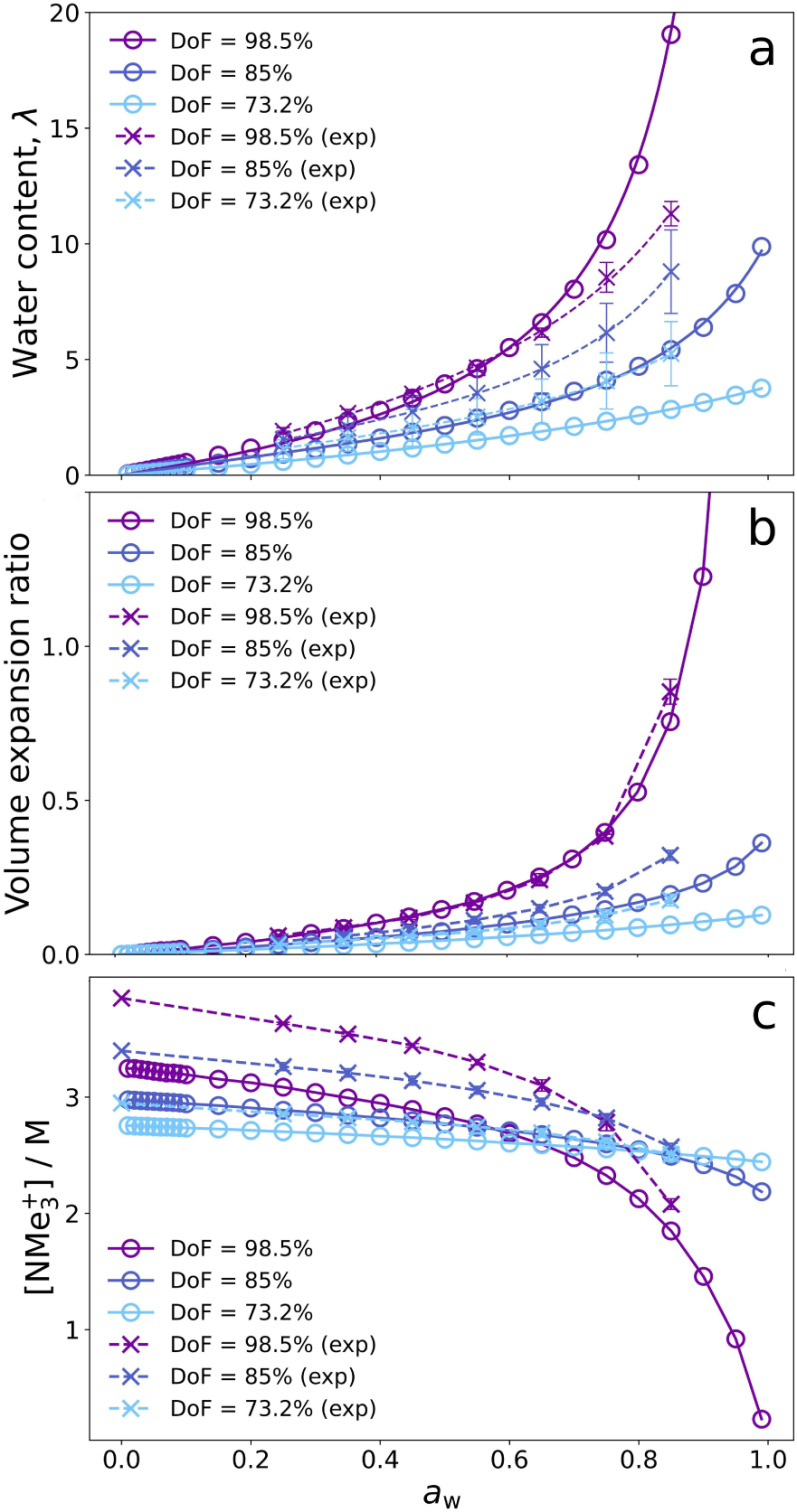
Model predictions (circles with solid lines) and experimental measurements^16^ (crosses with dashed lines) of the water content (a), volume expansion ratio (b), and ion concentration (c) as a function of the water activity of the vapor reservoir for polymers with varying degree of functionalization (DoF). The ion exchange capacities of the polymers are 2.69, 3.05, and 3.43 *mmol*/*g* for DoF = 73.2%, 85% and 98.5%, respectively. Water content is expressed as hydration number (number of water molecules per ion pair), volume expansion ratio as the increase in the volume relative to the dry volume, and ion concentration as the molar concentration of ion pairs.

At high water activity, *a*_*w*_, the predicted isotherms anticipate higher water absorption than that observed in experiments. We believe that this discrepancy is primarily due to incomplete equilibration in the experiments at high relative humidity. To support this hypothesis, we show in Figure S2 the mass of the membrane exposed to humid air over time, measured by a Quartz Crystal Microbalance (see ref 16). Initially, the mass increases rapidly and equilibrates at an apparent plateau, but then continues to rise at a much slower rate. This gradual increase persists for at least 2 days, until finalization of the experiment, at which point the membrane mass is 4.4% higher than the initial equilibrated value (see Figure S2). Furthermore, we observe that the materials partially dissolve when submerged in liquid water, over a time window of around 2 to 3 days. This observation qualitatively aligns with the model predictions for water content at *a*_*w*_ > 0.95, which are compatible with the dissolution of the polymer chains in water.

Another possible source of discrepancy is the observation that ion exchange membranes show higher water absorption in contact with liquid water than with a vapor reservoir of equal water activity *a*_*w*_, a phenomenon known as Schroder’s paradox.^46^ This effect has been attributed to kinetic factors^47^ as well as differences in the morphology of the polymer at the interface.^21,48,49^ Note that our model cannot capture either of these phenomena since it does not access kinetically trapped metastable states, nor does it consider the polymer-reservoir interface. Furthermore, the model does not explicitly describe the water reservoir; vapor and liquid water reservoirs with equal *a*_*w*_ are indistinguishable.

Finally, a minor contribution to the discrepancies at high humidity may arise from the simplicity of the model, which neglects some free-energy contributions that could become significant in this region. Specifically, the model does not consider the connectivity of the chains. For this reason, the entropic penalty due to the adoption of specific chain configurations is absent. Note that this contribution is expected to be minor for long chains.^50^

The model accurately predicts several properties of the material that depend on its level of hydration. For example, Figure 2b,c shows the volume expansion ratio (defined as (*V*_*wet*_ – *V*_*dry*_)/*V*_*dry*_ where *V*_*wet*_ and *V*_*dry*_ are the volumes of the hydrated and dry polymer respectively) and the molar concentration of ion pairs as a function of the water activity for different degrees of functionalization. The plots show good agreement with experiments. The volume expansion ratio increases with the water activity as a result of the expansion of the polymer when it absorbs water. The increase in the volume is approximately ideal, see Figure S3, which is in agreement with experimental results for the same system.^16^ The effect of the degree of functionalization follows the same trend as the water content, that is, the expansion is milder for lower DoF. Ion concentration, in turn, exhibits an interesting behavior (see Figure 2c). At low humidity, ion concentration is higher for polymers with higher DoF. At high humidity, this trend reverses because of the greater volume expansion experienced by polymers with higher DoF. This inversion is directly explained by the volume expansion; before becoming hydrated, the polymers with higher DoF have higher ion concentrations, but as water activity increases, polymers with higher DoF expand more, leading to a sharper decrease in ion concentration.

In the next section, we address how various polymer design parameters often explored experimentally in the literature affect the absorption behavior according to the model predictions. We focus on the effect of the nature of the counterions, as well as the length and hydrophobicity of the side chains.

### Effect of the Polymer Design

We calculate absorption isotherms for systems with different monovalent counterions. Specifically, we vary systematically the type of beads that represent the counterion using the options available in the Martini 3 force field,^36^ i.e., the “Q” series from Q1 to Q5. In this series, ions are more kosmotropic and exhibit stronger interactions with water in going from Q1 to Q5. To give a rough reference, Q1 might represent a hexafluorophosphate, and Q5 a chloride. The calculated isotherms are presented in Figure 3a. Our results show that water content increases with the kosmotropic character of the counterions. This finding is consistent with experimental data in the literature for both anion^13,21^ and cation conducting membranes.^51,52^ For instance, Kusoglu et al.^21^ demonstrated that the water content of poly­(aryl piperidinium) membranes increases along the counterion series I^–^, Br^–^, Cl^–^, OH^–^. A similar result was observed for polysulfone membranes.^13^

**Figure 3:**
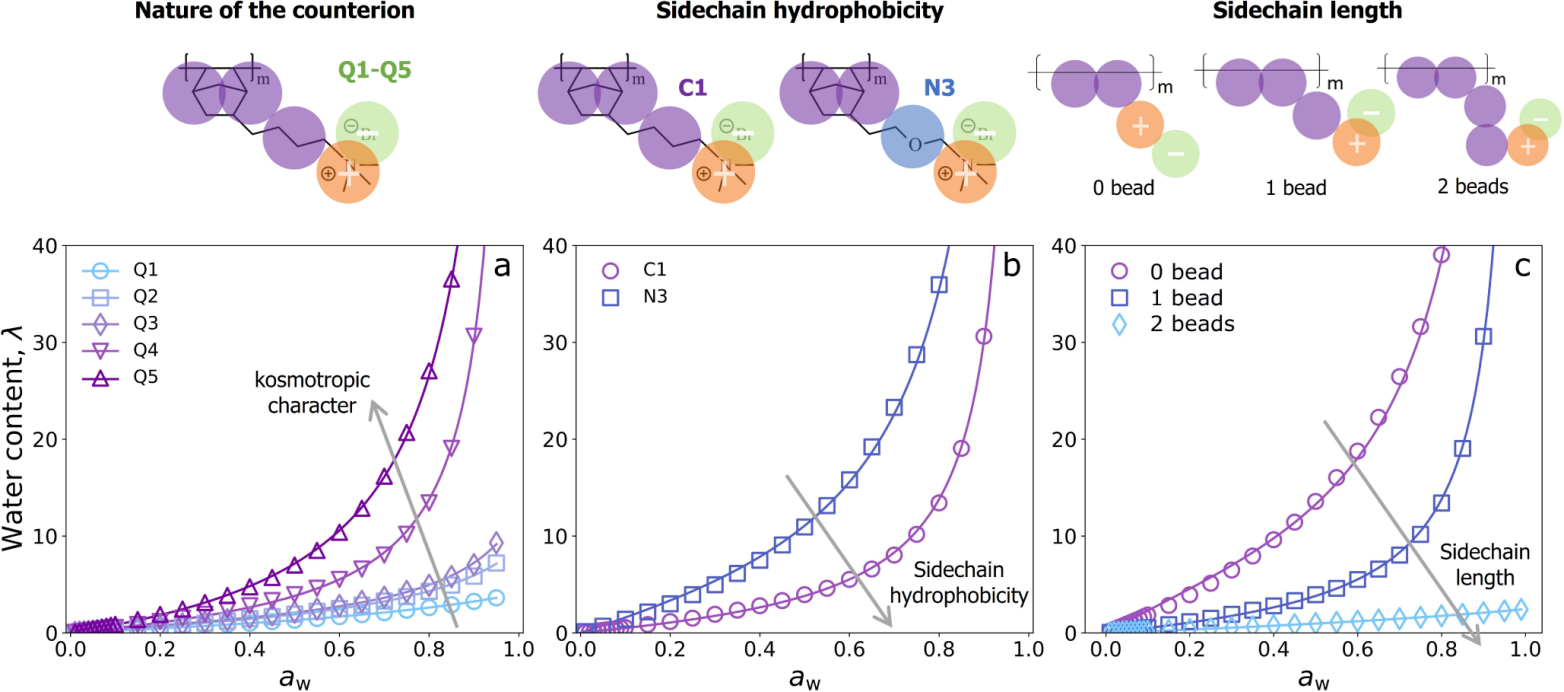
Model predictions of the water absorption isotherms for polymers with different counterions (a), side chain hydrophobicity (b), and side chain length (c). Water content is expressed as hydration number (number of water molecules per ion pair).

To investigate how the hydrophobicity of the polymer side chain affects water absorption, we consider a polymer with an ethylene glycol (EO) unit replacing the original alkyl chain. Figure 3b shows the calculated isotherms for both polymers. The polymer with the EO unit exhibits significantly higher water absorption compared to the one with the alkyl side chain. This result aligns well with previous experimental findings, which indicate that increasing the hydrophobicity of the chains leads to a decrease in the water content.^53–56^ For example, Qaisrani et al. found that replacing alkyl chains with ethylene glycol units increases the water content of aromatic ionomers.^54^ It is worth noting that, in some cases, the effect of the hydrophobicity of the side chain is less straightforward.^57,58^

Finally, we vary the length of the alkyl side chain by changing the number of coarse-grain beads it comprises. The predicted isotherms (Figure 3c) indicate that as the side chain length increases, the water content decreases. This effect of the length of the side chain is well documented in the literature for both anion and proton exchange membranes.^53,59–62^

### Energy of Interbead Attractions and Molecular Depiction of the Absorption Process

To gain a deeper understanding of the results presented in the previous sections, it is helpful to examine the absorption process at the molecular level. Figure 4a shows the energy of the short-range attractions between beads as a function of water content, for a polymer with a degree of functionalization (DoF) equal to 98.5%. These interactions are classified into two main groups: cohesive and water-attractive. Cohesive interactions are those present in the system before water absorption occurs, that is, attractive interactions between polymer and ion beads (backbone–backbone, backbone-side chain, backbone-ions, side chain-side chain, side chain-ions, ions–ions). Water-attractive interactions are those that draw water from the vapor reservoir into the condensed system, including attractive interactions between water and polymer beads, water and ion beads, and the self-interaction of the water beads.

**Figure 4:**
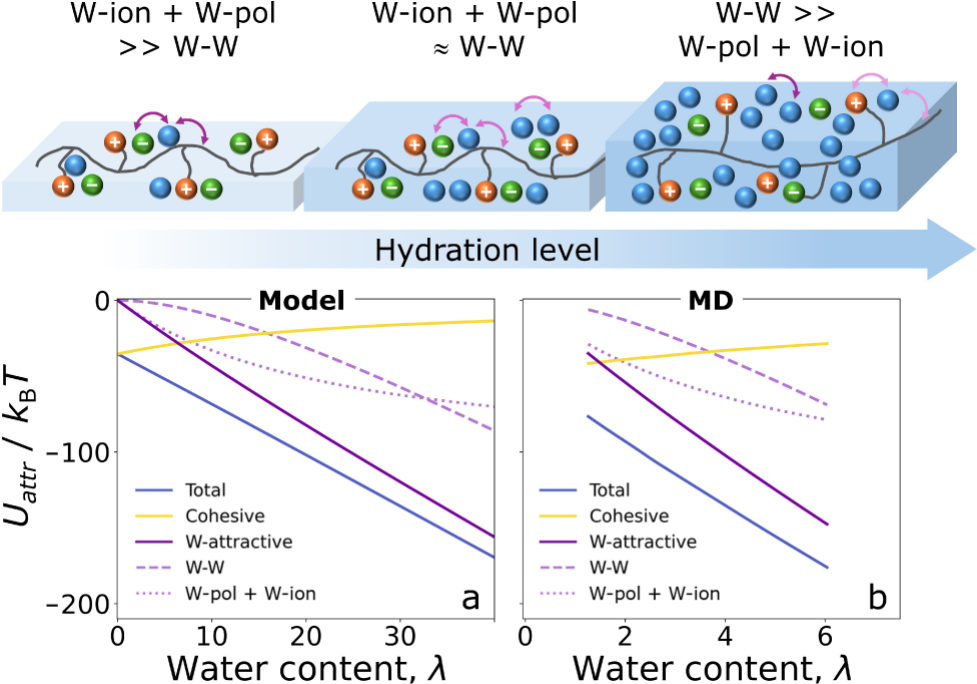
Energy per ion pair of the Lennard-Jones short-range attractions between different parts of the system as a function of water content (expressed as hydration number) calculated by the model (a) and from molecular dynamic simulations (b, see calculation details in SI and ref 63). Cohesive interactions are defined as all the attractive interactions between polymer and ion beads. W-attractive interactions are all the interactions that attract water from the reservoir into the system, including attractive interactions between water and polymer beads, water and ion beads, and water beads self-interaction. At the top, we present a schematic drawing of the hydration process at a molecular level derived from the model predictions of the energy of the short-range attractions versus water content.

The energy of cohesive interactions increases monotonically with water content, while that of water-attractive interactions decreases (see Figure 4a). In other words, water absorption is favored by water-attractive interactions and opposed by the cohesive interactions between polymer and ion beads. This framework allows us to analyze the effect of polymer design on water content by examining how the balance between cohesive and water-attractive interactions is affected by the polymer’s chemical structure. For example, increasing the degree of functionalization of the polymer results in a higher ion–water attractive energy, which leads to an increase in the water content. Similar reasoning applies to the effect of the counterion nature. Changing the chemistry of the side chain length also affects the water-attractive interactions; the ethylene glycol unit will have stronger attractive interactions with water than the alkyl chain, leading to higher water content. On the other hand, increasing the length of the side chain results in an increase in the cohesive energy, thereby reducing the water content.

Figure 4a shows that at low water content, the water-attractive interactions are completely dominated by polymer–water and ion–water interactions (see dotted purple lines), with the latter being the most significant (see Figure S4). As water content increases, water–water interactions begin to play a more important role, competing with water–polymer and water–ion interactions. Finally, at high water content, the energy of water–polymer and water–ion attractions levels off, and water-attractive interactions become entirely dominated by water–water attractions. This behavior agrees with the current understanding of the hydration process of ion exchange membranes, supported by several experimental and simulation results.^14,16,64–67^ In general, water is described to be primarily coordinated with the ions at the early stages of the hydration isotherm, and then mainly coordinated with other water molecules at later stages. The process is schematically represented in the top panel of Figure 4.

The qualitative behavior of the energy of short-range interactions predicted by the model coincides with energy calculations based on all-atom molecular dynamics (MD) simulations of the same system conducted previously by our group,^63^ see Figure 4b. All the key features observed in the model predictions are also present in the MD calculations. However, the MD water content is approximately 4 times lower than that of the model. The discrepancy arises in part because the molecular models used in the MD simulations and in this work were parametrized by comparing experimental measurements of water content obtained from two different experimental setups. The MD molecular model was parametrized to reproduce the behavior of bulk membranes whose water content was measured by Dynamic Vapor Sorption (DVS). The experimental data against which this model was tested correspond to Quartz Crystal Microbalance (QCM) measurements of the water content of thin membranes (with thickness in the range of ∼100–200 nm depending on the level of hydration).^16^ The water content can be significantly affected by the membrane thickness^68^ as well as the general experimental setup.

Note that the QCM water content is around 2 times higher than the DVS measurements. Hence, the quantitative discrepancy between MD calculations and this model cannot be solely ascribed to differences in the reference experimental data. Some quantitative disagreement is expected given the different molecular and spatial resolutions of MD simulations and our model. For instance, our model overlooks the membrane heterogeneity at the nanometric scale. It is worth mentioning that the water content in the MD simulations was fixed to the experimental values rather than predicted for each water activity of the reservoir.

The agreement between the predictions of this model and MD simulations also extends to the coordination number of the ions, see Figure S5. In the model, the fraction of ions that form ion pairs with counterions is 1 at low water content and decreases as water content increases, though this effect becomes significant only at high water content. This result is qualitatively in agreement with MD calculations of the coordination number of bromide anions with respect to quaternary ammonium cations; see Figure S5.

### Water Volume Fraction Governs the Thermodynamics of Water Absorption

In addition to the energy of the short-range interactions, other free-energy contributions play a significant role in the absorption process. Figure 5a,b shows the contributions that are significant to the absorption process as a function of water activity for polymers with DoF = 98.5% (a) and DoF = 85% (b). These contributions are categorized into “favorable” contributions (they drive water into the system and decrease with increasing water content) and “unfavorable” contributions (they prevent water from entering the system and increase with increasing water content). The favorable contributions include the water-attractive short-range interactions and a minor contribution of the mixing entropy of the species, especially at high water activity. The unfavorable contributions consist of the cohesive short-range interactions, the hard-sphere repulsions, and the water chemical potential term (see eq 1). The remaining contributions are negligible relative to the total free energy and approximately invariant with the water content. In particular, the free energy due to ion-pair formation has a negligible contribution to the hydration process, since ion pairs are disrupted only at high water content where the free energy is predominantly governed by water contributions, see Figure S5.

**Figure 5:**
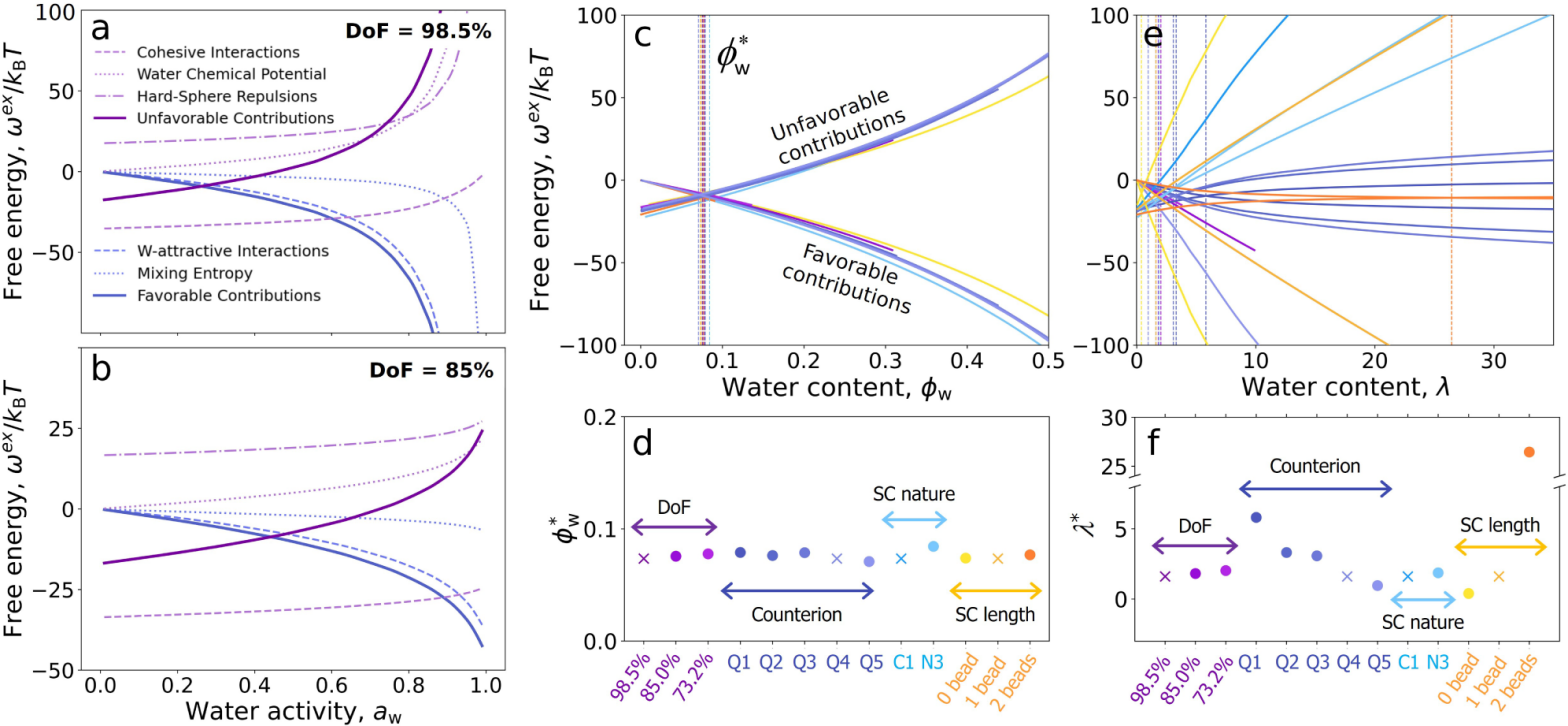
Free energy contributions per ion pair as a function of the water activity calculated by the model for DoF = 98.5% (a) and 85% (b). Favorable and unfavorable free energy contributions per ion pair as a function of water volume fraction (c) and hydration number (e) for all the polymer designs considered in this work. Vertical dashed lines indicate the volume fraction $\phi_{w}^{*}$ or hydration number λ* at which favorable and unfavorable contributions have the same value. $\phi_{w}^{*}$ (d) and λ* (f) for all the polymer designs. The colors in panels c, d, e and f indicate a specific polymer design, i.e., polymers with varying DoF are shown in shades of purple, polymers with varying counterion in shades of blue, polymers with varying side-chain nature in shades of light blue and polymers with varying side-chain length in shades of orange. The crosses correspond to the same design, i.e., a polymer with DoF = 98.5%, ion bead = Q3, and a side chain with one C1 bead.

The free-energy contributions for polymers with DoF = 98.5% and 85% shown in Figure 5a,b exhibit similar features, but at different water activities. The same features are also observed for all the other polymer designs considered in this work, see Figure S6. The free-energy contributions versus water activity plots for polymers with lower water content show similar trends at higher water activities. For example, the favorable and unfavorable contributions intersect at *a*_*w*_ ≈ 0.25 for DoF = 98.5% and at *a*_*w*_ ≈ 0.45 for DoF = 85%. Interestingly, at these points, both polymers show a similar value of hydration number (λ  ≈ 1.5), suggesting that the thermodynamics of the absorption process may be governed by the water content.

To test this hypothesis, Figure 5c,e shows favorable and unfavorable contributions as a function of the water content expressed as both the volume fraction of water (the volume occupied by the water beads divided by the total occupied volume) and the hydration number (water-to-ion pair molar ratio) for all polymer designs considered in this work. Remarkably, the free-energy contributions collapse in a master curve when plotted against the water volume fraction, but not against the hydration number. This collapse is also absent when the free-energy contributions are plotted versus the water-to-polymer mass ratio (commonly referred to as ″Water Uptake″) and the water-to-monomer molar ratio, see Figure S7. These results indicate that the volume fraction of water governs the thermodynamics of the hydration process, regardless of the polymer chemical design.

To better understand the collapsed behavior of the free-energy contributions, we focus on the water volume fraction at which the favorable and unfavorable contributions are equal, denoted $\phi_{w}^{*}$ (marked with vertical dashed lines and color-coded by polymer design in Figure 5c, and plotted in Figure 5d using the same color code). Although the definition of this landmark is arbitrary, it serves as a useful reference for characterizing the behavior of free-energy contributions. Note that $\phi_{w}^{*}$ is ∼0.08 for all polymers (Figure 5e), while the analogs for the hydration number (λ*, Figure 5f) and other measures of the water content (Figure S7) vary with polymer design.

Note that other measures of the water content can be used to effectively collapse the free energy contributions when a subset of specific design parameters is considered. For example, Figure 5f shows that λ* remains constant for polymers with equal backbone structure but different degrees of functionalization, DoF. This suggests that for polymers with the same chemical design but varying DoF, the thermodynamics is apparently governed by the hydration number, even though the underlying controlling variable is the volume fraction of water. The same is true for other measurements of the water content, see Figure S7.

The water volume fraction at which unfavorable and favorable contributions equilibrate, $\phi_{w}^{*}$ , can be related to the molecular description of the hydration process by examining the energy of short-range attractions. Figure 6a shows the energy due to water-attractive and cohesive interactions as a function of the water volume fraction for a polymer with 98.5% degree of functionalization. The point at which water–water interactions begin to contribute significantly to water-attractive interactions coincides with $\phi_{w}^{*}$ . This observation holds for all polymer designs, as illustrated in Figure 6b.

**Figure 6:**
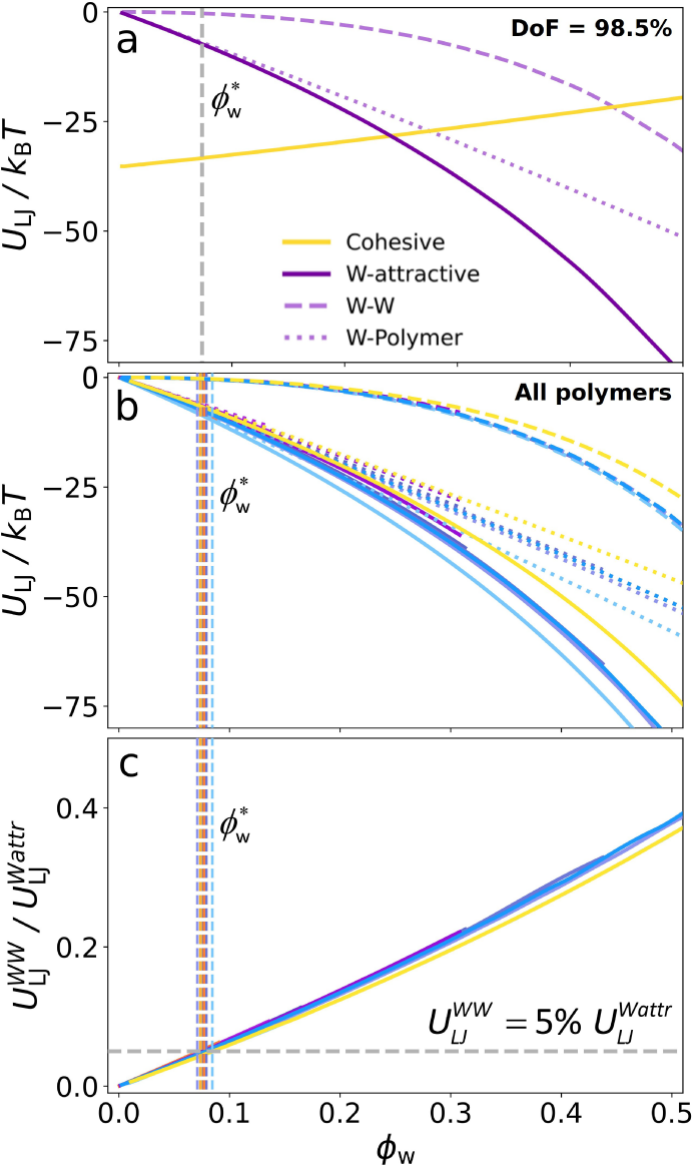
Energy per ion pair of the short-range interactions as a function of the water volume fraction, ϕ_*w*_, for the polymer with 98.5% degree of functionalization (a) and for all the polymers (b). Ratio between the energy due to water self-interaction and the total water-attractive interactions as a function of the water volume fraction, ϕ_*w*_, for all the polymers (c). Vertical dashed lines indicate the water volume fraction at which favorable and unfavorable contributions present the same value, $\phi_{w}^{*}$ . In panels b and c, the colors follow the same color code as in Figure 5 to indicate polymer design.

To further support this statement, Figure 6c shows the ratio between the energy of the water self-interaction and that of the total water-attractive interactions for all polymer designs. All curves collapse into a universal behavior, indicating that the importance of water–water interactions relative to the total water-attractive interactions is also governed by the water volume fraction. At $\phi_{w}^{*}$ , water–water interactions represent 5% of the total water-attractive interactions. In other words, ϕ_*w*_ ≈ 0.08 is the point where water molecules begin to connect with each other (see schematic drawing in Figure 4). This is an important result that may have an impact on the relationship between the membrane hydration process and the ionic conductivity. In the next section, we address this matter along with other implications and the scope of the results of our model.

## Discussion

We showed that our model accurately predicts the hydration isotherms of polynorbornene-based anion exchange membranes, capturing the correct trends for polymers with different degrees of functionalization, counterion nature, side-chain length, and side-chain hydrophobicity. The model indicates that the hydration level of the membranes is dominated by the balance between hydrophilic and hydrophobic interactions within the material. More importantly, it reveals that the thermodynamics of the absorption process is governed by the water volume fraction; that is, the contributions to the free energy collapse in the same universal manner as a function of water volume fraction for any polymer design. This behavior extends to the relative importance of the interactions that draw water into the membrane (water–water, water–ion, and water–polymer attractions), suggesting that the process at a molecular level is also controlled by the water volume fraction. This leads to our main conclusion: the stages of the absorption process at the molecular scale are reached at the same water volume fraction (but not at the same hydration number or water uptake), regardless of the specific polymer design.

This finding has implications for the relationship between the hydration process and the ionic conductivity. The ionic conductivity of both cation and anion exchange membranes has been extensively studied, and different mechanisms have been proposed.^14,69–72^ In general, it is agreed that for ions to conduct through the membrane, a percolated water network must be established. According to percolation theory,^73,74^ the percolation of a network is governed by the relative volume occupied by the percolating element and the topology of the network. This implies that ion conductivity mainly depends on the water volume fraction in membranes with similar microscopic organization throughout the hydration process. This statement is supported by experimental measurements of the ionic conductivity of membranes, which often show a collapsed trend as a function of water volume fraction.^21,75,76^

The fact that both the hydration thermodynamics and the ionic conductivity are controlled by the volume fraction of water has important consequences on the development of better ion exchange membranes. If both processes are governed by the same property, it complicates the design of membranes with intrinsically higher conductivity at lower hydration levels. However, this double control is valid only for membranes with a similar organization at the nanoscale, with water networks that maintain their topological characteristics throughout the whole hydration process. This is not the case for many systems. In addition, different polymer designs could exhibit different organization at the nanoscale. For instance, membranes can be homogeneous^16^ or form water-rich and polymer-rich subdomains of different shapes such as lamellae^77^ or bicontinuous phases,^14^ depending on the polymer design. This may affect the universal control of water volume fraction over conductivity and hydration. Moreover, this interpretation assumes that ion conductivity is explained solely by the percolation of the water network. We have recently shown that ion and water networks percolate together based on results of our coupled-layer model^16^ and molecular dynamics simulations,^63^ but this might not be the case for some systems.

Although the double control of water volume fraction might not be universal, we believe that it provides a reasonable first approximation to evaluate whether a given membrane presents an intrinsically higher conductivity or if the boost is a mere effect of hydration. In that sense, our results suggest that the water volume fraction is the correct control parameter to assess this, and thus a better option to normalize the effect of hydration on conductivity in comparison to other usual parameters such as the hydration number or the mass water uptake. Moreover, this interpretation of the connection between hydration and conductivity sheds light on possible avenues to design better membranes. The most common strategy to achieve enhanced conductivity is to promote the emergence of nanomorphologies that facilitate the percolation of the water network. In general, most attempts have focused on generating or controlling the characteristics of water nanochannels^78^ through chemical design or processing.^79,80^ Other promising strategies aim to interconnect ions without the presence of water, reducing the dependence of the ionic conductivity on the percolation of the water network.^81^

The structure of the membrane at the nanoscale can also affect the water content. Our homogeneous model is intended to address the hydration process at a length scale larger than the spatial resolution required to observe the formation of water- and polymer-rich nanodomains. This approximation is based on the fact that membranes are macroscopically homogeneous, that is, they do not truly phase separate into distinct water- and polymer-rich regions at a macroscopic scale and hence form a single thermodynamic phase. However, the formation of nanodomains or aggregates can be coupled with the hydration process and thus affect it. Some membranes do not show these features,^68^ or the nanostructure does not change significantly throughout the hydration process,^82,83^ for which cases we believe the homogeneous approximation is suitable. In some cases, the entrance of water triggers a nanosegregation process^82^ or significantly changes the shape of the aggregates.^14^ In such cases, the homogeneous model will not capture how changes in the nanostructure of the membrane affect the absorption isotherms. In the future, we plan to formulate an inhomogeneous version of the model that can predict both the equilibrium water uptake and the explicit nanostructure of the membrane as a function of the water activity of the reservoir,^35^ to study the interplay between the two phenomena.

Finally, our model was tested for polynorbornenes of specific designs. Although we believe our conclusions could be extended to a broader chemical space, this needs to be contrasted. In that sense, the inhomogeneous model will also allow us to consider explicit polymer chains in order to assess more complex polymer structures. In addition, we will investigate how the conformational freedom of the chains and the presence of cross-links may affect the hydration process.

## Conclusions

In summary, we have presented a simple model to target the water absorption isotherms of polynorbornene anion exchange membranes. The model accurately predicts the water content versus water activity plots for polymers with various degrees of functionalization, as well as the effect of the counterion nature, side-chain hydrophobicity, and side-chain length. Additionally, the model’s predictions for other hydration-dependent properties, such as volume expansion ratio and ion concentration, are consistent with experimental measurements. The model was further validated by comparing the predicted energy of short-range attractions with results from molecular dynamics simulations.

The analysis of the energy of pairwise attractive interactions allowed us to describe the absorption process at the molecular level. Initially, water molecules interact exclusively with the ions. As water content increases, water–water interactions become increasingly significant, eventually dominating water–ion interactions at high water content. In addition, the model reveals the free energy contributions that are significant to the absorption process. These contributions are entirely governed by the water volume fraction, regardless of the polymer design. The water volume fraction at which the contributions that favor water uptake balance those that oppose it marks the point at which water molecules begin to connect to each other.

The universal control of water volume fraction over the water absorption process has implications for the design of membranes with intrinsically higher ionic conductivity. In particular, for membranes that exhibit an invariant network topology, the fraction of water can control both the conductivity and the hydration process, suggesting that decoupling these two phenomena could be challenging.

In the future, we plan to extend the model to consider inhomogeneities at the nanoscale. This will allow us to address how the hydration process is affected by the reshaping of the nanostructure with varying water activity. In addition, we plan to incorporate an explicit description of the polymer chains to expand the control over the polymer design and consider chain-correlation contributions to the hydration process.

## Supplementary Material



## Notes

Detailed description of the theoretical methods, long-term equilibration of the hydrated membrane (Figure S2), ideal behavior of the volume expansion of the membrane (Figure S3), water attractive interaction versus water content (Figure S4), detailed description of the molecular dynamics simulations, fraction of ions forming ion pairs versus water content (Figure S5), and favorable and unfavorable free energy contributions versus water activity and water content for all polymer designs (Figure S6 and S7) (PDF)
