# Supplementary material for: The Role of Water Volume Fraction on Water Adsorption in Anion Exchange Membranes
Source: Macromolecules. 2025 Sep 11;58(18):9972–82. doi: 10.1021/acs.macromol.5c01256 (PMC12461909; doi:10.1021/acs.macromol.5c01256)
Supplement: Supplementary file 1 [file ma5c01256_si_001.pdf]

Supporting Information: The Role of Water Volume Fraction on Water Adsorption in Anion Exchange Membranes

Gervasio Zaldivar,[†,‡,¶,§] Ruilin Dong,[§] Joan M. Montes de Oca,[‖] Ge Sun,[†,‡,¶,§] Riccardo Alessandri,[§,⊥] Christopher Arges,[#] Shrayesh N. Patel,[§] Paul F. Nealey,[*,§,‖] and Juan J. de Pablo[*,†,‡,¶,§,‖]

†*Department of Chemical and Biomolecular Engineering, Tandon School of Engineering, New York University, Brooklyn, NY, USA*

‡*Department of Computer Science, Courant Institute of Mathematical Sciences, New York University, New York, NY, USA*

¶*Department of Physics, New York University, New York, NY, USA*

§*Pritzker School of Molecular Engineering, University of Chicago, Chicago, IL, USA*

‖*Materials Science Division, Argonne National Laboratory, Lemont, IL, USA*

⊥*Department of Chemical Engineering, KU Leuven, Celestijnenlaan 200J, 3001 Leuven, Belgium*

#*Applied Materials Division, Argonne National Laboratory, Lemont, IL, USA*

E-mail: nealey@uchicago.edu; depablo@uchicago.edu

Theoretical methods

Free energy density

We consider a system with fixed volume V and temperature $T = 298K = (k_B\beta)^{-1}$ (where k_B is the Boltzmann constant) comprising N_p polyelectrolyte chains each composed of n_p total monomers, of which n_+ are charged. To maintain electroneutrality, the system contains $n_- = N_p n_+$ counterions. We consider that the system is in contact with a water vapor reservoir with fixed water activity, i.e. fixed water chemical potential, μ_w, although we do not take into account the interface between the hydrated polyelectrolyte and the water reservoir. The proposed free energy density for the described system is:

$$\begin{aligned}\frac{\beta\Omega(N_p,V,T,\mu_w)}{V} &= \beta\omega(\rho_p,T,\mu_w) = \sum_{i=w,\rho,-}\rho_i\left[\ln\left(\rho_i v\right)-1\right] + \frac{\beta g}{2}\sum_{ij}\frac{N_i}{V}\frac{N_j}{V}\epsilon_{ij} \\ &+ \frac{\nu\rho_{tot}^2\left(4-3\nu\rho_{tot}\right)}{(1-\nu\rho_{tot})^2} + n_+\rho_p\left[f\ln f + (1-f)\ln(1-f)\right] \\ &+ n_+\rho_p\left[f\beta\mu_+^0 + (1-f)\beta\mu_{+-}^0\right] + \beta\rho_-\mu_-^0 + 2fn_+\rho_p u^B\left(\frac{1}{\varepsilon}-\frac{1}{\varepsilon_w}\right) - \beta\mu_w\rho_w \quad (1)\end{aligned}$$

Note that the free energy density in Equation 1 is a thermodynamic potential that is canonical for the polymer chains and counterions and grand canonical for the water.

The first term of Equation 1 accounts for the free energy related to the mixing entropy of the species in the system, where ρ_i is the number density of species i ($i = p$ for polymer chains, $-$ for counterions, and w for water molecules) and v^{-1} is the reference number density (v is the volume of the beads, see Molecular Model section).

The second term is the energy due to short-range attractions between the beads that form the species present in the system, where g is a prefactor that accounts for the integrated spatial dependence of the interaction potential, $\frac{N_i}{V}$ is the number density of the bead of type i, and ϵ_{ij} is the Lennard-Jones parameter that governs the strength of the interaction between beads of type i and j. More details are available in the Molecular Model section.

The third term is the energy of the interbead hard-sphere repulsions given by the Carnahan-Starling equation of state (EOS),[1,2] where ρ_{tot} is the total number density of beads, given by:

$$\rho_{tot} = \rho_w + n_b\rho_p + n_+\rho_p \tag{2}$$

where n_b is the number of beads in a polymer chain (see Molecular Model section) and $n_+\rho_p$ is the number density of counterions which is fixed by the value of ρ_p to ensure electroneutrality. Note that the Carnahan-Starling EOS assumes that all beads have the same volume v. In this work, v is equal to 43 Å^3 which is 4 times the hard-sphere volume of a water molecule[3] and around 2/3 of that of a butane molecule[3] or four methylene groups.[4] The choice of this value for v is explained further in the Molecular Model section below.

The fourth term represents the entropy associated with the possibility for an ionic monomer to form an ion pair with a counterion or remain unpaired. This possibility is considered through an association chemical reaction between the ionic monomers and the counterions.[5–8] The association reaction is represented by:

$$C^+ + A^- \rightleftharpoons [C^+A^-] \quad k_{as} = \frac{[C^+A^-]c^0}{[C^+][A^-]} \tag{3}$$

where k_{as} is the equilibrium constant of the association reaction, $[C^+]$, $[A^-]$ and $[C^+A^-]$ represent the molar concentrations of the cation, anion and the ionic pair respectively, and c^0 is the reference concentration ($1M$). In this term, f is the average fraction of cations that do not form ion pairs. In this work, $k_{as} = 156.4$.[8]

Note that the total number density of counterions (both free in solution or forming ion pairs) is given by $n_+\rho_p$ (see Equation 2) while we denote ρ_- (see the first term of Equation 1) the number density of counterions that are free in solution, $\rho_- = fn_+\rho_p$. Similarly, the total number density of cations is also given by $n_+\rho_p$, while the number density of free cations is $\rho_+ = \rho_- = fn_+\rho_p$.

The fifth and sixth terms in Equation 1 are the contributions of the standard chemical

potentials of the species involved in the association equilibrium, where μ^0_+, μ^0_- and μ^0_{+-} are the standard chemical potentials of the free cation, free anion and ionic pair, respectively. Although the standard chemical potential of all species should be taken into account in the free energy density, only those that are involved in the association equilibrium have thermodynamic relevance.

The seventh term in Equation 1 accounts for the energy change involved in the transfer of both ions from an infinitely diluted water solution to the dielectric medium. In Equation 1, ε and ε_w are the relative dielectric constants of the system and pure water respectively, and u^B is given by:

$$u^B = \frac{q^2}{8\pi\varepsilon_0 a_i} \tag{4}$$

where q is the charge of the ions, $q = |e|$, ε_0 is the vacuum electric permittivity, and a_i is the radius of the ions, $a_i = (\frac{3}{4\pi}v^{1/3})$. The dielectric constant of the system is modeled by the average of the dielectric constant of the components weighted by their volume fractions, ϕ_i, relative to the total volume fraction, ϕ_{tot}:

$$\varepsilon = \sum_{i=w,p,-} \frac{\phi_i}{\phi_{tot}}\varepsilon_i = \frac{(\rho_w + n_+\rho_p)\varepsilon_w + n_b\rho_p\varepsilon_p}{\rho_{tot}} \tag{5}$$

The second equality in Equation 5 results from considering the dielectric constant of solvated anions (either forming an ion pair with the cations or free in solution) equal to that of water. We also make use of the facts that $\phi_{tot} = v\rho_{tot}$ and that the volume fractions of water, counterions and polymer chains are $v\rho_w$, $vn_+\rho_p$ and $vn_b\rho_p$, respectively.

Finally, the last term of Equation 1 is the free energy associated with the chemical potential of water, which makes the thermodynamic potential grand canonical with respect to the water molecules.

Minimization of the free energy density

To find the equilibrium state of the system for a given set of values of the natural variables of the system (ρ_p, μ_w and $T = 298K$), we minimize the free energy density, ω, with respect to the fraction of ions that do not form ion pairs, f, and the number density of water, ρ_w. Note that the number density of the free counterions ρ_- is not an independent variable since its value is fixed by f, ρ_p and n_+.

The minimization of ω with respect to ρ_w leads to:

$$v\rho_w = \exp(\beta\mu_w - \beta u_{vdw} - \beta u_{HS} - \beta u_{Born}) \tag{6}$$

In Equation 6, each term represents an interaction present in the system that affects the equilibrium water density. The first term is the water chemical potential that is fixed by the vapor pressure of the reservoir. The second term accounts for the energy of the short-range attractive interactions, given by:

$$u_{vdw} = g \sum_i \epsilon_{wi} \frac{N_i}{V} \tag{7}$$

where i represents each type of bead present in the system (water, counterion, and polymer beads, see Molecular Model), ϵ_{wi} is the Lennard-Jones parameter for the interaction between bead i and water, and $\frac{N_i}{V}$ is the number density of bead i. The third term represents the hard-sphere repulsion and is given by:

$$u_{HS} = \frac{8\rho_{tot}v + 9(\rho_{tot}v)^2 + 3(\rho_{tot}v)^3}{(1 - \rho_{tot}v)^3} \tag{8}$$

The fourth term is related to the Born energy and is given by:

$$u_{Born} = \frac{2fn_+\rho_p u^B}{\varepsilon\rho_{tot}} \left(\frac{\varepsilon_w}{\varepsilon} - 1\right) \tag{9}$$

The minimization of ω with respect to f yields:

$$-\beta(\mu^0_{+-} - \mu^0_{+} - \mu^0_{-}) = \ln\left[\frac{1-f}{f^2 n_+ \rho_p v}\right] \tag{10}$$

Considering that the standard Gibbs free energy of the association reaction given by Equation 3 is $\Delta G^0_{as} = \mu^0_{+-} - \mu^0_{+} - \mu^0_{-}$ and that the number density of free counterions is $\rho_- = f n_+ \rho_p$, Equation 10 can be rearranged into:

$$\exp\left(-\beta \Delta G^0_{as}\right) = K^0_{as} = \left[\frac{1-f}{f\rho_- v}\right] \exp\left[2u^B\left(\frac{1}{\varepsilon_w} - \frac{1}{\varepsilon}\right)\right] \tag{11}$$

where $K^0_{as} = k_{as}(c^0 N_A v)^{-1}$, and N_A is the Avogadro number.

Equations 6 and 11 are coupled non-linear equations that are solved by numerical methods for each given set of natural variables of the system, i.e. the temperature, T (298K in this work), the number density of the polymer, ρ_p, and the chemical potential of the water, μ_w. This procedure leads to the number density of water and the fraction of ions that do not form an ion pair. In the section Criteria for the Thermodynamic Equilibrium, we discuss how we find the equilibrium structural properties of the membranes for a given chemical potential of water.

Molecular Model

We designed the molecular model based on the Martini 3 description.[9] The polymer chains are composed of beads, each representing approximately four heavy (non-H) atoms, see Figure 1 in the main text. In Martini 3, different functional groups are represented by different types of beads that are classified according to their hydrophobicity and relative affinity. In our model, the polynorbornene ring is represented by two C2 martini beads and the alkyl side chain is formed by a variable number of C1 beads, while the ethylene glycol unit is represented by one N3 bead. In charged monomers, the side chains present one Q3 bead at the end representing the terminal trimethylammonium (TMA) group, while in neutral

monomers the last C1 bead of the alkyl chain is replaced by a X2 bead. In all calculations, the counterion is Br^-, represented by a Q4 bead. Finally, to represent water molecules, we selected the Martini 3 regular water bead, denoted W, which represents 4 combined water molecules. The polymer chains are formed by 236 monomers, and the number of charged and neutral monomers is given by the degree of functionalization, DoF.

In Martini 3 simulations, beads present attractive nonbonded pairwise interactions that are governed by parameters ϵ_{ij} and σ_{ij} through a Lennard-Jones potential. For this work, we only take the attractive branch of such potential, given by:

$$u_{ij}^{LJ} = g_{ij}(|\mathbf{r} - \mathbf{r}'|)\epsilon_{ij} \tag{12}$$

where:

$$g_{ij}(|\mathbf{r} - \mathbf{r}'|) = -\left(\frac{\sigma_{ij}}{|\mathbf{r} - \mathbf{r}'|}\right)^6 \tag{13}$$

if $|\mathbf{r}-\mathbf{r}'| > 2^{1/6}\sigma_{ij}$ and 0 otherwise. In this work, both σ_{ij} and ϵ_{ij} were taken directly from the Martini 3 force field. Specifically, we used beads with the same value for $\sigma_{ij} = \sigma = 0.47nm$.

Since our model is homogeneous, the spatial dependence of the potential, $g_{ij}(|\mathbf{r} - \mathbf{r}'|)$, can be integrated over the spatial coordinates to reach the expression of the second term of Equation 1 where g accounts for the integrated spatial dependence of the interactions, given by:

$$g = \frac{2^{3/2}}{3}\pi\sigma^3 \tag{14}$$

We emphasize that we are only considering the attractive branch of the Lennard-Jones potential, and modeling the repulsions through a hard-sphere equation of state. Furthermore, we are restricted to consider beads of the same size, while Martini 3 molecular descriptions usually comprise a combination of beads of different sizes. It is hence expected that the choice of beads in the context of this model may be adjusted with respect to that of a Martini 3 molecular dynamics simulation. For instance, in this case we decided to use Q3 for the TMA group instead of the usual Q2, and larger, slightly more hydrophobic beads for

the polynorbornene structure.

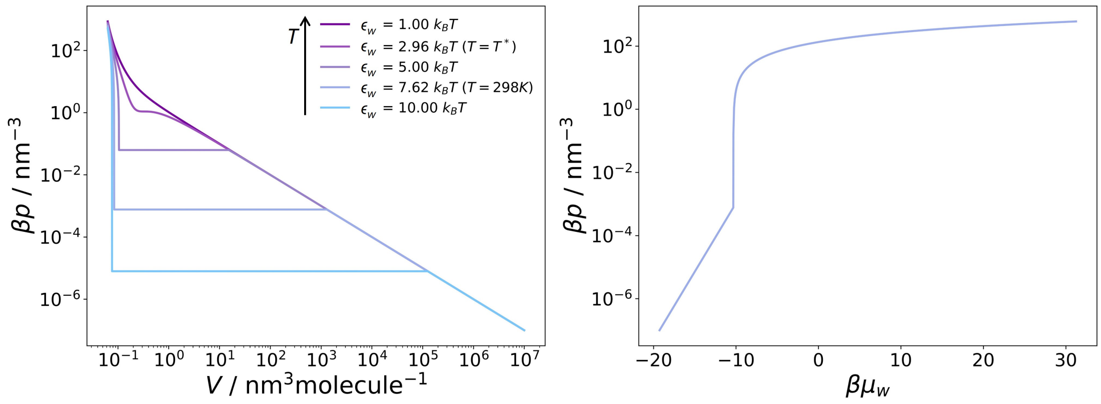

Figure S1: Equilibrium vapor pressure of water, p, as a function of the volume per molecule (left) and water chemical potential, μ_w (right), predicted by the model for different temperatures.

The model allows us to calculate the phase diagram of water as a function of the pressure and the volume, by defining $\rho_p = 0$. We performed such calculation with two purposes. The first one is to adjust the value of the hard-sphere volume of the beads, v, to reproduce the equilibrium water vapor pressure at room temperature. The second one is to find the relationship between the pressure of the water vapor reservoir, p, and the water chemical potential, μ_w. Note that the vapor pressure of the water reservoir (p) is referred to as p_{vap} in the main text.

Figure S1 (left panel) shows the equilibrium vapor pressure of the reservoir as a function of the volume per water molecule, for $v = 43$Å and various temperatures. Note that each curve corresponds to a different value of ϵ_w expressed in units of k_BT. Although at first order, ϵ_w is inversely proportional to T, second-order dependencies might be significant. Therefore, we decided not to explicitly indicate the exact temperature for each curve, except for room temperature.

The volume was chosen so the vapor pressure for $\epsilon_w = 7.62k_BT$ (the value given by the Martini for $T = 298K$) is equal to the experimental value, $p = 3142Pa$ or $\beta p = 0.246nm^{-3}$.[10] The predicted liquid number density is $47.4nm^{-3}$ which is $\sim$ 40% higher than the exper-

imental value. The predicted value of the critical pressure is $\beta p = 1.089 nm^{-3}$ which is around 44% of the experimental value, $\beta p = 2.48 nm^{-3}$. We believe the accuracy of this prediction is good enough considering the simplicity of the water model and that we do not freely adjust the molecular parameters. The only exception is the hard-sphere bead volume that was in principle adjusted to reproduce the vapor pressure at room temperature. Note, however, that the resultant value is in line with the hard-sphere volume of the functional groups represented by the bead, i.e. 43Å is ~ 4 times the hard-sphere volume of a water molecule[3] and around 2/3 of that of a butane molecule[3] or four methylene groups.[4]

Finally, this calculation allows us to relate the vapor pressure of the water reservoir to the water chemical potential, μ_w, see Figure S1. This relationship effectively translates the water activity of the reservoir, p/p^* (where p^* is the saturated pressure), to the actual natural variable of the thermodynamic potential, μ_w.

Criteria for the Thermodynamic Equilibrium

We formulated the model using a semi-grand canonical ensemble with N_p, V, T and μ_w as natural variables. For a wet system in contact with the water reservoir at a given vapor pressure p, we seek the equilibrium value of ρ_p, which is equivalent to finding the equilibrium volume V of a system with constant N_p at fixed pressure p.

We followed the procedure described in Ref. 2. In equilibrium, the internal pressure of the system, p_i is equal to the external pressure p:

$$\beta p_i = \left. \frac{\partial \beta \Omega[N_p, V, T, \mu_w]}{\partial V} \right|_{N_p, T, \mu_w} = \beta p \tag{15}$$

which can be rewritten as the following condition:

$$\left. \frac{\partial \beta \tilde{\Omega}[N_p, p, T, \mu_w]}{\partial V} \right|_{N_p, T, \mu_w} = 0 \tag{16}$$

where $\tilde{\Omega}$ is the Legendre transform of Ω with respect to the volume, given by:

$$\beta\tilde{\Omega}[N_p, p, T, \mu_w] = \beta\Omega[N_p, V, T, \mu_w] + \beta p V \tag{17}$$

$\beta\tilde{\Omega}$ is hence the proper thermodynamic potential to evaluate the relative thermodynamic stability of systems with different volumes V and equal external pressure p, number of polymer chains N_p and water chemical potential μ_w.

Since the system is homogeneous, the criterion described by Equation 16 can be rewritten as:

$$\left.\frac{\partial \beta\tilde{\omega}\rho_p^{-1}}{\partial \rho_p^{-1}}\right|_{N_p,T,\mu_w} = 0 \tag{18}$$

where $\tilde{\omega}$ is $\tilde{\Omega}/V$. Note that $\tilde{\omega}$ can be also interpreted as the excess semi-grand canonical free energy density with respect to the water reservoir, ω^{ex}:

$$\beta\omega^{ex} = \frac{\beta\Omega(N_p, V, T, \mu_p) - \beta\Omega^R}{V} \tag{19}$$

where $\Omega^R = \Omega(N_p = 0, V, T, \mu_p) = -\beta p V$.

In sum, for each given water vapor pressure, we calculate $\tilde{\omega}$ as a function of ρ_p and then find the minimum of $\tilde{\omega}\rho_p^{-1}$ with respect to ρ_p^{-1}. The corresponding value of ρ_p represents the equilibrium hydrated system.

Equilibration of the hydrated membrane

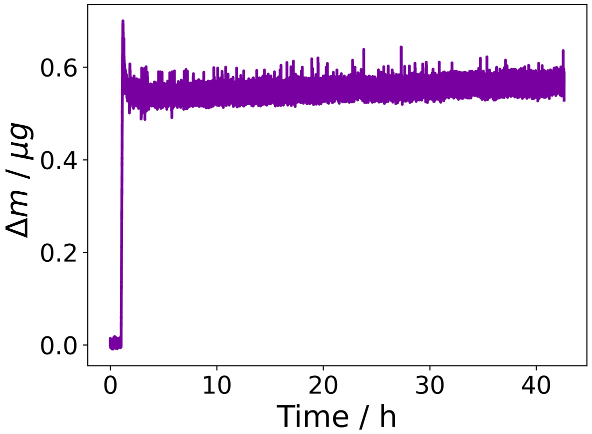

Figure S2: Mass increase as a function of time for a polymer with 98.5% degree of functionalization at 0.9 water activity. The graph shows continuous water absorption behavior at slow speed after initial apparent equilibration.

Ideal behavior of the volume expansion

Figure S3 shows the volume expansion ratio as a function of the water content for varying degrees of functionalization. The volume increases approximately linearly with the number of water molecules normalized by the number of ion pairs (i.e., the hydration number, λ), which indicates negligible excess volume.

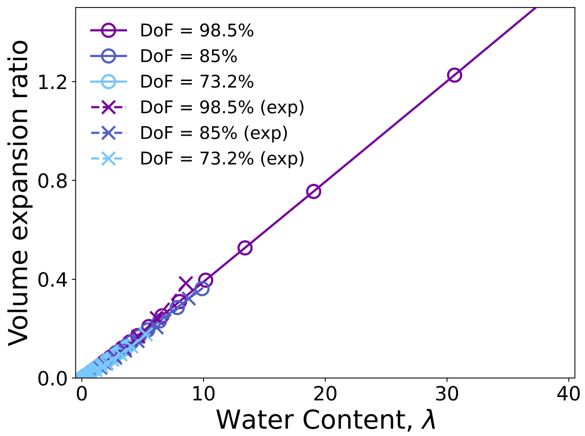

Figure S3: Volume expansion ratio as a function of the water content expressed as hydration number, λ, for different degrees of functionalization. Solid lines are model predictions and dashed lines are experimental measures from Ref. 11

Detail of water attractive interactions

Figure S4 shows the energy due to the attractive interactions between water and ion beads (W-Ion), and between water and polymer beads (W-Pol). The energy of W-Ion interactions represents around two thirds of the combined W-Ion and W-Pol interactions. This ratio is fixed for all hydration levels because W-Ion and W-Pol interaction energies are proportional to the number density of ions and polymer beads respectively, which quantities are related through the stoichiometric relationship between ions and polymer molecules.

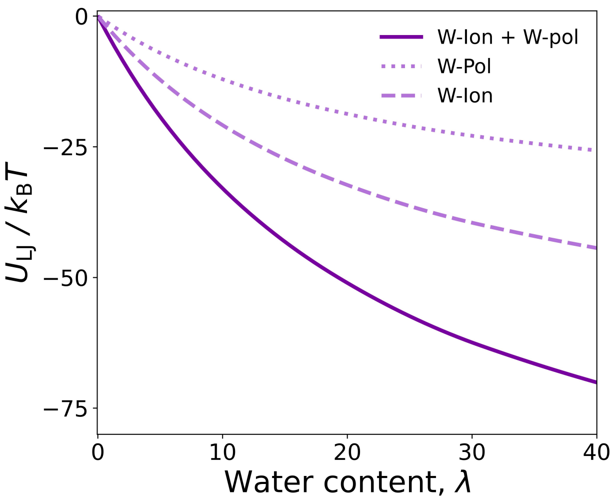

Figure S4: Energy due to water-ion and water-polymer attractive interactions as a function of the water content expressed as hydration number for a polymer with 98.5% degree of functionalization.

Molecular Dynamics Simulations

All molecular dynamics (MD) simulations were conducted using the same all-atom force field as in our previous work.[12] Specifically, the parameters for bromide ions and quaternary ammonium groups were derived from the Canongia Lopes & Padua (CL&P) force field,[13] while all other parameters were taken from the All-Atom Optimized Potentials for Liquid Simulations (OPLS-AA) force field.[14] For hydrated systems, the SPC/E water model was employed. The GROMACS[15] simulation package was used to perform all MD simulations. The equations of motion were integrated using the leap-frog algorithm with a 1 fs timestep. Nonbonded interactions were calculated with a cutoff distance of 12 Å, and the smooth particle-mesh Ewald (SPME) method was applied for long-range electrostatics. During initial equilibration in NPT simulations, the v-rescale thermostat and Berendsen barostat were used. Subsequent NPT simulations utilized the Nosé-Hoover thermostat and Parrinello-Rahman barostat. Production simulations in the NVT ensemble were carried out with the Nosé-Hoover thermostat.

Energy contribution calculations

The interaction energies between different species were calculated using GROMACS MD simulations. Initially, distinct groups were defined based on the species of interest. The interaction energy was computed by separating the contributions into Coulombic and Lennard-Jones (LJ) terms, considering only short-range interactions up to the cutoff distance specified earlier. Long-range electrostatic interactions were handled using the smooth particle-mesh Ewald (SPME) method, which efficiently computes reciprocal space contributions. It is important to note that while the SPME method accounts for long-range electrostatics, these contributions cannot be decomposed to provide explicit long-range interactions between individual groups. Similarly, the Lennard-Jones interactions were truncated at the cutoff distance for short-range calculations, while long-range van der Waals forces were approximated using the standard dispersion correction applied to the overall system energy. Given that our focus is on the local interactions between ions, water molecules, and polymer chains, the exclusion of long-range interaction contributions between specific groups does not affect the accuracy of our conclusions. The localized short-range interactions dominate the behavior of the system within the scope of this study.

Fraction of ions forming ion pairs

Panel a of Figure S5 shows the fraction of trimethyl ammonium (TMA) cations forming ion pairs with Br^- anions, $1 - f$, predicted by the model, while panel b shows the coordination number of the N atom from TMA cations forming part of the first shell of Br^- anions, determined by MD simulations.[12] Although the model only considers the possibility of forming ion pairs, neglecting multiparticle coordination, the model predictions and the MD results show qualitative agreement. MD simulations show that cations and anions are on average always coordinated for all hydration levels, although the Br-N coordination number decreases from ∼4.5 to ∼2.5. In turn, the model predicts that the fraction of ions forming ion pairs is close

to 1 and significantly decreases only at high levels of hydration.

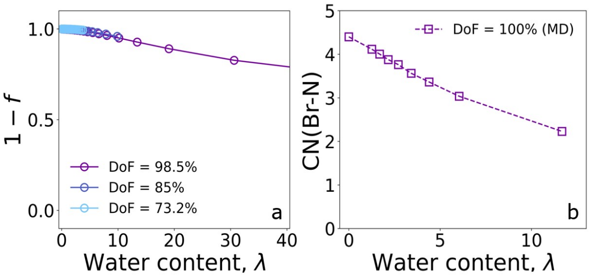

Figure S5: Average fraction of TMA ions forming ion pairs with Br^-, $1 - f$, predicted by the model (a) and number of N atoms in the first coordination shell of Br^- ions, CN(Br-N) in MD simulations taken from Ref. 12 (b) as a function of the hydration number.

Free energy contributions

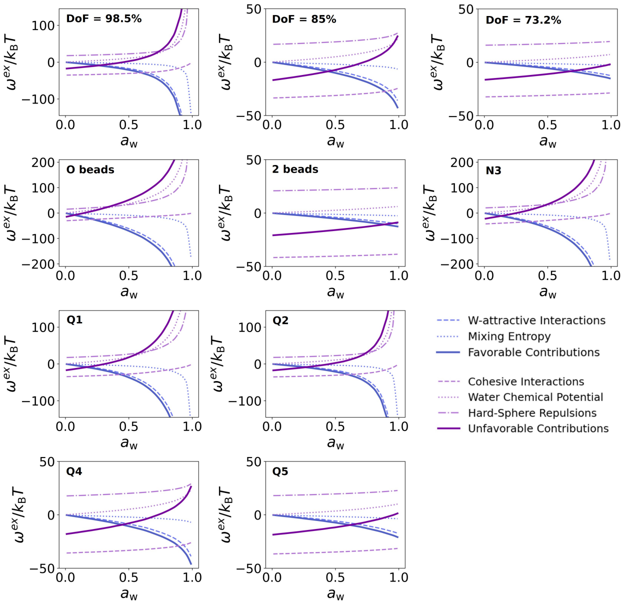

Figure S6: Free energy contributions per ion pair as a function of the water activity calculated by the model for all the polymers considered in this work

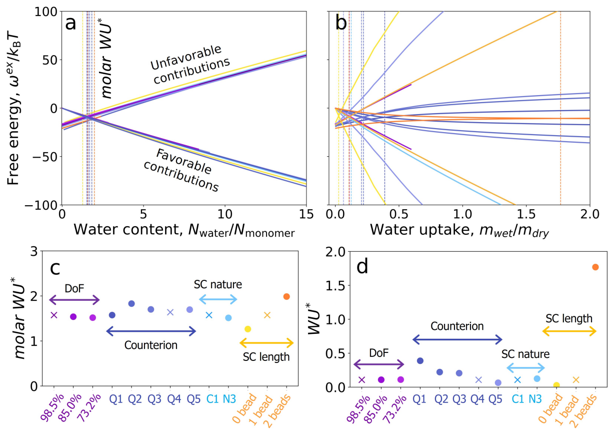

Figure S7: Favorable and unfavorable free energy contributions per ion pair as a function of water-to-monomer molar ratio (a) and wet-to-dry mass ratio (b). Vertical dashed lines indicate the point at which favorable and unfavorable contributions have the same value. Water-to-monomer molar ratio, $molarWU^*$, (a) and wet-to-dry mass ratio, WU^*, (d) at which favorable and unfavorable contributions have the same value. The crosses correspond to the same design, i.e. a polymer with DoF = 98.5%, ion bead = Q3, and a side chain with none C1 beads.

References

(1) Carnahan, N. F.; Starling, K. E. Equation of State for Nonattracting Rigid Spheres. *The Journal of Chemical Physics* **1972**, *51*, 635–636.

(2) Missoni, L. L.; Upah, A.; Zaldívar, G.; Travesset, A.; Tagliazucchi, M. Solvent Isotherms and Structural Transitions in Nanoparticle Superlattice Assembly. *Nano Letters* **2024**, *24*, 5270–5276.

(3) Gogonea, V.; Băleanu-Gogonea, C.; Osawa, E. Solvent hard sphere diameter from van der Waals volume A statistical analysis of computed and solubility determined solvent diameters. *Journal of Molecular Structure: THEOCHEM* **1998-06-15**, *432*, 177–189.

(4) Ben-Amotz, D.; Willis, K. G. Molecular hard-sphere volume increments. *The Journal of Physical Chemistry* **1993-07**, *97*, 7736–7742.

(5) Kramarenko, E. Y.; Erukhimovich, I. Y.; Khokhlov, A. R. The Influence of Ion Pair

Formation on the Phase Behavior of Polyelectrolyte Solutions. *Macromolecular Theory and Simulations* **2002**, *11*, 462–471.

(6) Zaldivar, G.; Tagliazucchi, M. Layer-by-Layer Self-Assembly of Polymers with Pairing Interactions. *ACS Macro Letters* **2016**, *5*, 862–866.

(7) Friedowitz, S.; Salehi, A.; Larson, R. G.; Qin, J. Role of electrostatic correlations in polyelectrolyte charge association. *The Journal of Chemical Physics* **2018**, *149*, 163335.

(8) Debais, G.; Tagliazucchi, M. Two Sides of the Same Coin: A Unified Theoretical Treatment of Polyelectrolyte Complexation in Solution and Layer-by-Layer Films. *Macromolecules* **2022**, *55*, 5263–5275.

(9) Souza, P. C. T. et al. Martini 3: a general purpose force field for coarse-grained molecular dynamics. *Nature Methods* **2021**, *18*, 382–388.

(10) Wagner, W.; Pruß, A. The IAPWS Formulation 1995 for the Thermodynamic Properties of Ordinary Water Substance for General and Scientific Use. *Journal of Physical and Chemical Reference Data* **2002-06-07**, *31*, 387–535.

(11) Montes de Oca, J. M.; Dong, R.; Zaldivar, G.; Sun, G.; Wang, Z.; Patel, S. N.; Nealey, P. F.; de Pablo, J. J. IEC-Independent Coupling between Water Uptake and Ionic Conductivity in Anion-Conducting Polymer Films. *Macromolecules* **2025**, *58*, 6134–6148.

(12) Wang, Z.; Sun, G.; Lewis, N. H. C.; Mandal, M.; Sharma, A.; Kim, M.; de Oca, J. M. M.; Wang, K.; Taggart, A.; Martinson, A. B.; Kohl, P. A.; Tokmakoff, A.; Patel, S. N.; Nealy, P. F.; de Pablo, J. J. Water-mediated ion transport in an anion exchange membrane. *Nature Communications* **2025**, *16*, 1099.

(13) Canongia Lopes, J. N.; Deschamps, J.; Pádua, A. A. H. Modeling Ionic Liquids Using

a Systematic All-Atom Force Field. *The Journal of Physical Chemistry B* **2004**, *108*, 2038–2047.

(14) Jorgensen, W. L.; Maxwell, D. S.; Tirado-Rives, J. Development and Testing of the OPLS All-Atom Force Field on Conformational Energetics and Properties of Organic Liquids. *Journal of the American Chemical Society* **1996**, *118*, 11225–11236.

(15) Abraham, M. J.; Murtola, T.; Schulz, R.; Páll, S.; Smith, J. C.; Hess, B.; Lindahl, E. GROMACS: High performance molecular simulations through multi-level parallelism from laptops to supercomputers. *SoftwareX* **2015**, *1-2*, 19–25.
